# Supplementary material for: Ion currents through Kir potassium channels are gated by anionic lipids
Source: Nat Commun. 2022 Jan 25;13:490. doi: 10.1038/s41467-022-28148-4 (PMC8789855; doi:10.1038/s41467-022-28148-4)
Supplement: Supplementary file 1 — Supplementary Information [file 41467_2022_28148_MOESM1_ESM.pdf]

Supplementary Materials

Ion currents through Kir potassium channels are gated by anionic lipids.

Jin *et al.*

Figures

Supplementary Figure 1. Kir Sequence alignment and ACMA assay.
Supplementary Figure 2. Crystal structures show the L124M mutant retains structural integrity.
Supplementary Figure 3. Single channel recordings of L124M show a preference for the fully open state.
Supplementary Figure 4. Comparative PMFs at the Leu124 and Tyr132 collars
Supplementary Figure 5. Ions only pass the Leu124 collar when the aperture has sufficiently widened.
Supplementary Figure 6. The *gauche* rotamer of Tyr57 blocks lipid entering the fenestrations
Supplementary Figure 7. MTS-alkyl derivatization.
Supplementary Figure 8. Normalised data from liposomal fluorescence flux (ACMA) assays.
Supplementary Figure 9. HMM analysis of single channel recordings of C119-alkylated KirBac3.1
Supplementary Figure 10. Aliphatic chain occupancy affects pore aperture in C119-derivatised channels.
Supplementary Figure 11. Anionic lipids preferentially interact with the KirBac3.1 channel.

Tables

Supplementary Table 1. Crystallographic Structure Determination and Refinement.
Supplementary Table 2. Summary of Molecular Dynamics Simulations.
Supplementary Table 3. Summary statistical data on fluorescence change for functional ACMA assays
Supplementary Table 4. Summary rates data for ACMA assays.

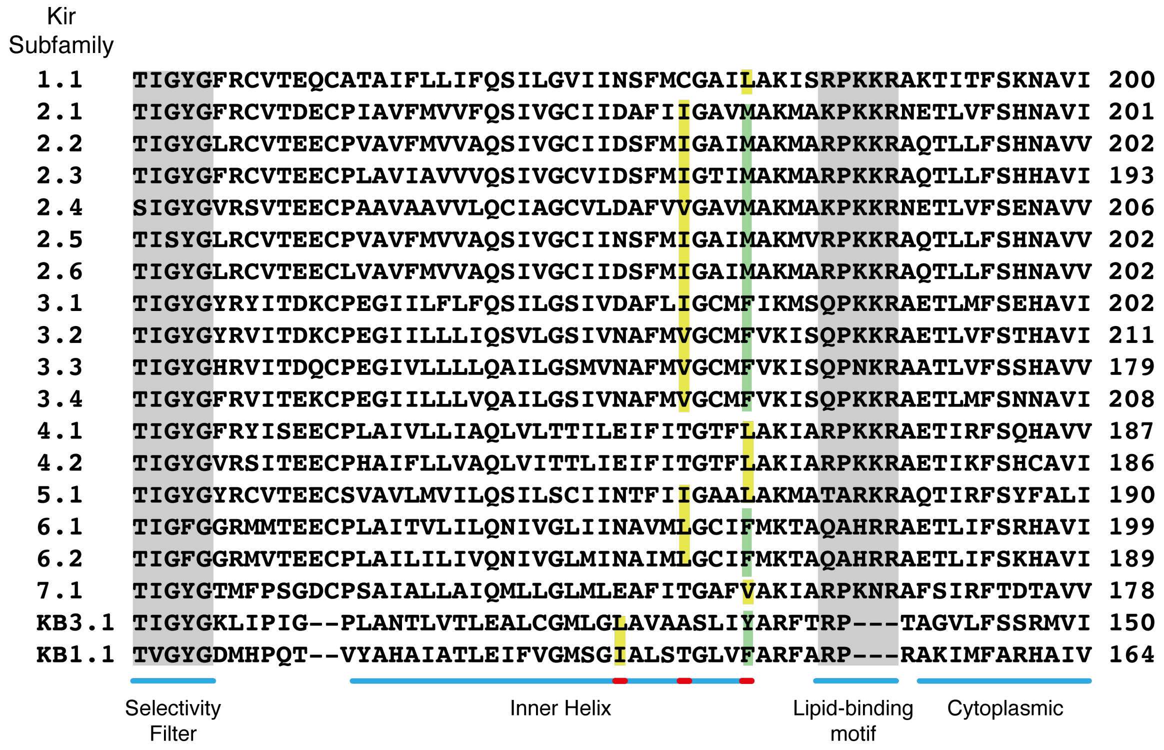

Supplementary Figure 1. **Kir Sequence alignment and ACMA assay.** Partial sequence alignment of human Kir subfamily members against the prokaryotic homologues KirBac1.1 (*Burkholderia pseudomallei*) and KirBac3.1 (*Magnetospirrilum magnetotacticum*). Labelled blue underscoring shows the sequence-to-structural alignment, with red segments indicating the inward-facing residues, each a helix-turn apart, lining the conduction pathway. The selectivity filter and basic lipid binding motif are shaded grey. Green shading indicates the residue constricting the cytoplasmic opening at the helix bundle crossing (the conventional site of the ‘gate’) and yellow the inward facing branched aliphatic residues – all have only one potential constriction site, except Kir5.1, which has two.

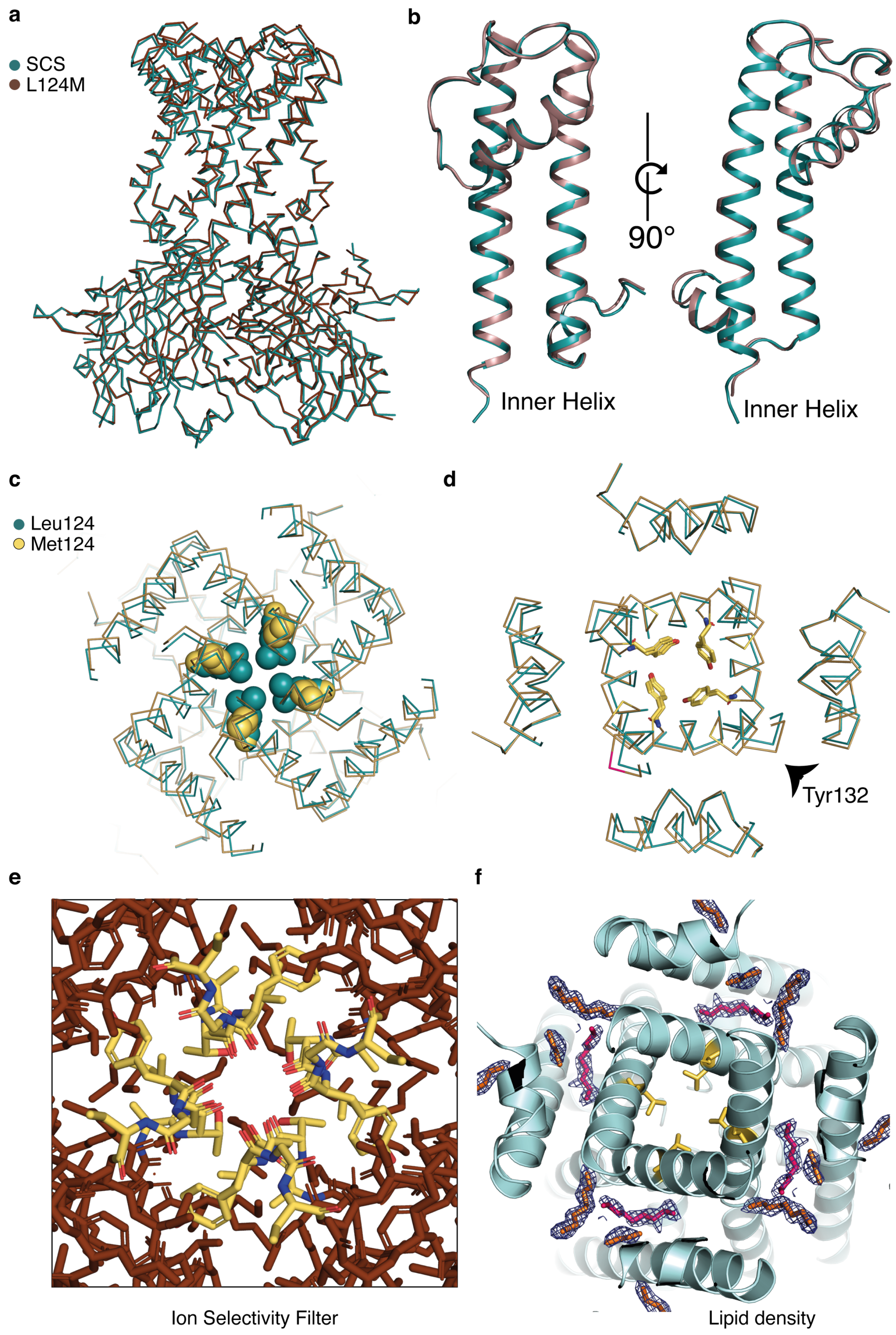

Supplementary Figure 2. **Crystal structures show the L124M mutant retains structural integrity**. (**a**) Ca overlay of the entire polypeptide backbones of the KirBac3.1-SCS (cyan) and L124M (SCS-L124M; brown). (**b**) Orthogonal views of a single subunit of KirBac3.1-SCS superposed on L124M. (**c**) cross-sectional slice of the overlay at Leu124 shows that, despite different side chains at residue 124, the Ca backbone does not significantly widen. (**d**) Cross-sectional slab of the overlay at the helix bundle constriction Tyr132. (**e**) The selectivity filter (TVGYG) in the L124M structure is conserved, with all selectivity filter carbonyls pointing inwards and coordinating ions (not shown). (**f**) Several fragments of lipid or detergent are built into the structure; a 2.4 Å sharpened (B factor -50 Å^2) (2|F_o|-|F_c| electron density map is contoured at 1 σ (dark blue mesh).

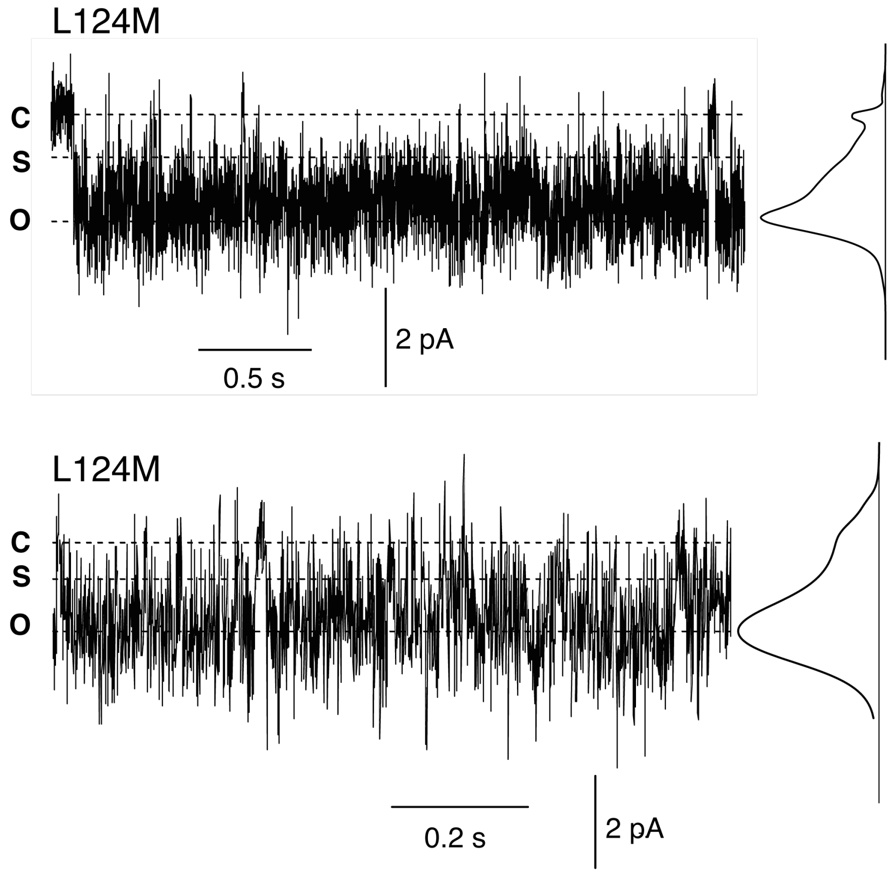

Supplementary Figure 3. Single channel recordings of L124M show a preference for the fully open state. Single channel recordings of the L124M mutant of KirBac3.1 at -60 mV (top) and -70 mV (bottom), filtered at 500 Hz. Open (O), closed (C) and partially open substate (S) levels determined by Hidden Markov Models are indicated by horizonal dashed lines. The maximum-likelihood amplitude histograms generated by Hidden Markov Models are aligned with the recordings.

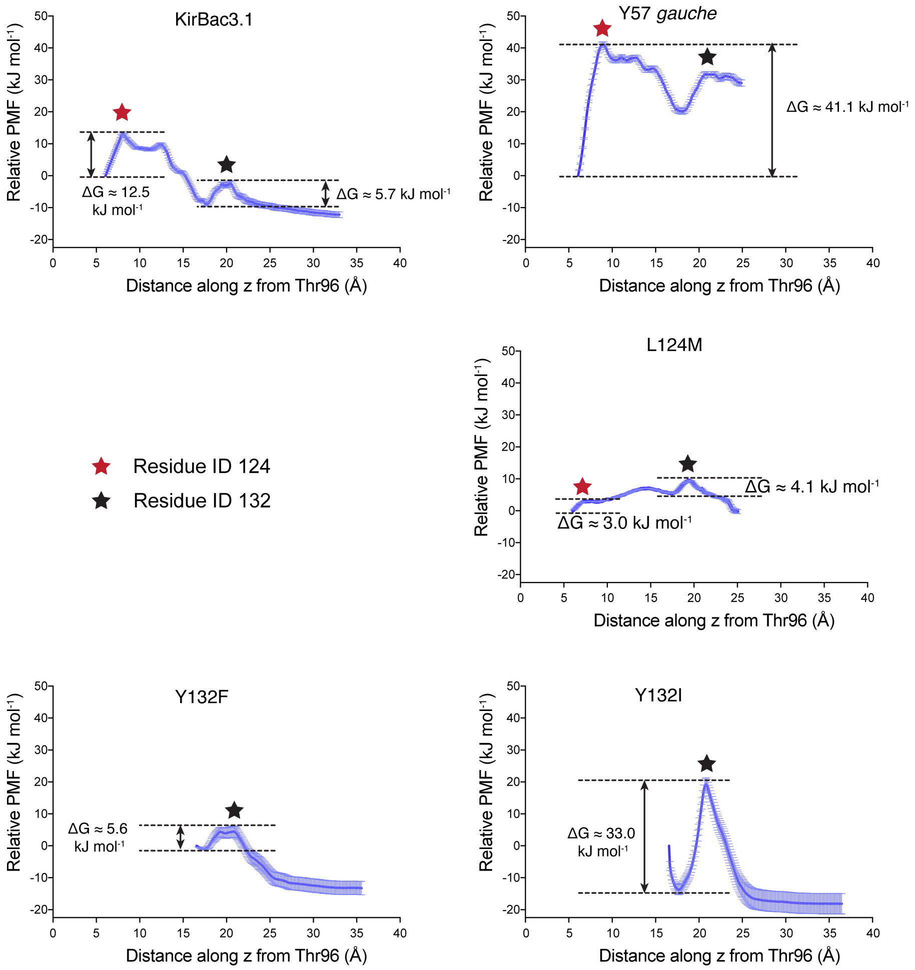

Supplementary Figure 4. Comparative PMFs at the Leu124 and Tyr132 collars. This shows the free energy barrier faced by a permeating K^+ at Leu124 collar and/or Tyr132 collar, in point mutants and in channels with the fenestration-blocking rotamer of Tyr57. The potential of mean force along the molecular 4-fold axis of a wild type KirBac3.1 structure is shown as a function of the distance between a K^+ cavity ion 'pulled' along the *z*-axis and the centre of mass of the four Thr96 sidechains (the global reaction coordinate (see Figure 1a)). Uncertainties, estimated via Bayesian bootstrap analysis with 200 bootstraps and a tolerance of 10^{-6}, are depicted as vertical lilac bars.

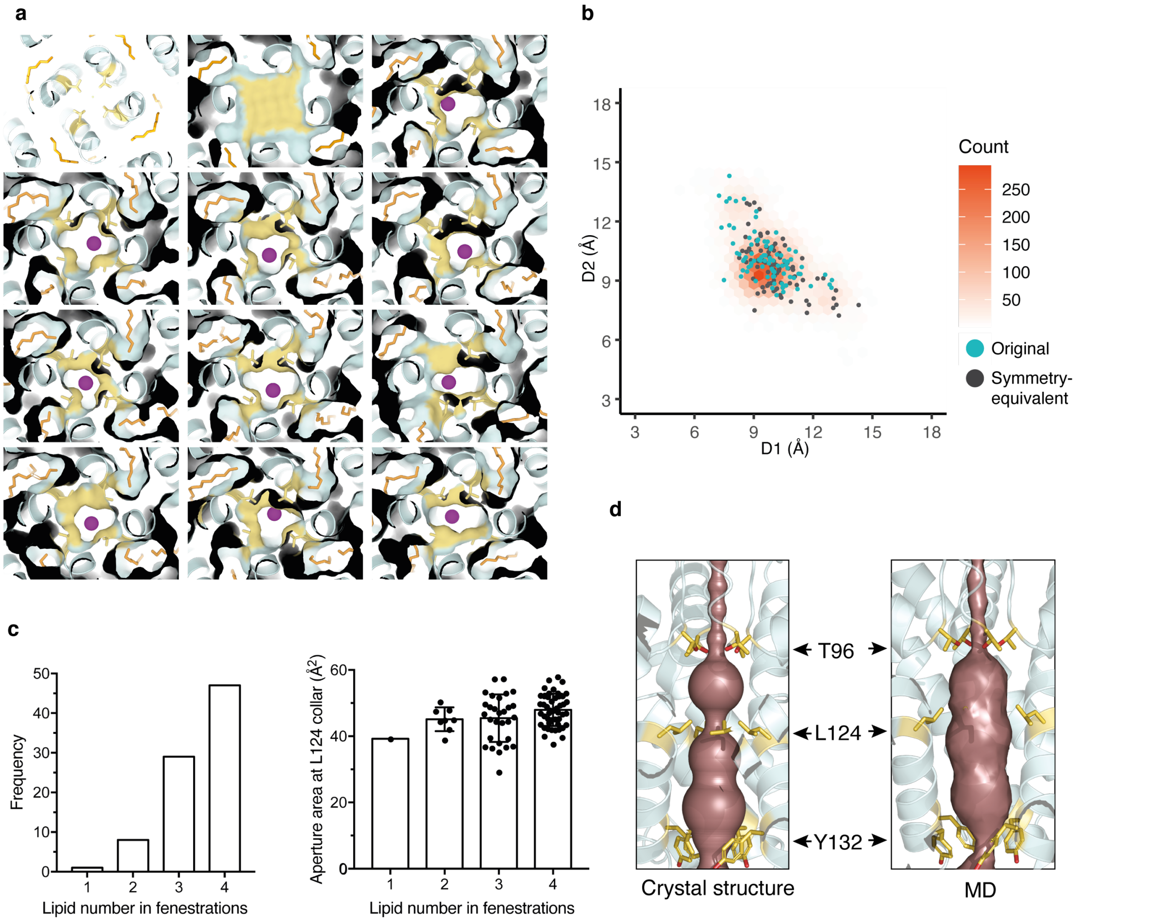

Supplementary Figure 5. Ions only pass the Leu124 collar when the aperture has sufficiently widened. (**a**) Cross-sectional slice through the KirBac3.1 pore (cyan) at the Leu124 collar of 10 randomly selected structures with a K^+ ion passing the collar. The accessible molecular surface demonstrating occlusion or opening up of the conduction pathway at the Leu124 collar. The left and middle panels in the top row are reference slabs from the crystal structure, slightly enlarged over Fig. 2a. Side chains and surface of Leu124 are coloured yellow, lipidic alkyl chains orange, and potassium depicted as purple spheres. (**b**) Hexagonal bin plot of diagonal distances, D1 vs D2 (see Fig 2d for definition) for structures in which an ion is passing through the Leu124 collar extracted from unrestrained MD. Symmetry equivalent pairs are included in the count and representation. Structures were distributed into hexagonal bins on the basis of D1-D2 couplets. The count in each bin is described by a colour key, with dark orange representing the greatest number of structures and white the least. The predominant central peak is as in Fig 2d and reflects a mean pore diameter of approximately 9 Å. Using data from short simulations, each corresponding to ~490 ps either side of the Leu-Cδ 'plane', the couplets of structures at the precise moment of passing the plane are plotted as cyan dots. The symmetry-equivalent D2-D1 couplets are shown as black dots. This illustrates the range of aperture size K^+ is able to permeate. (**c**) Analysis of lipid occupancy and aperture area (mean±SD from a total of 85 independent conduction events; n=85) at the Leu124 collar for permeation events. (**d**) Comparative internal cavity profiles for the crystal structure (Leu124 occludes the conduction path) and a representative MD conducting structure (Leu124 is engaged by lipid). The conduction pathway dimensions are output from HOLE (solid surface). The key difference between the structures is the release of the constriction at the Leu124 collar.

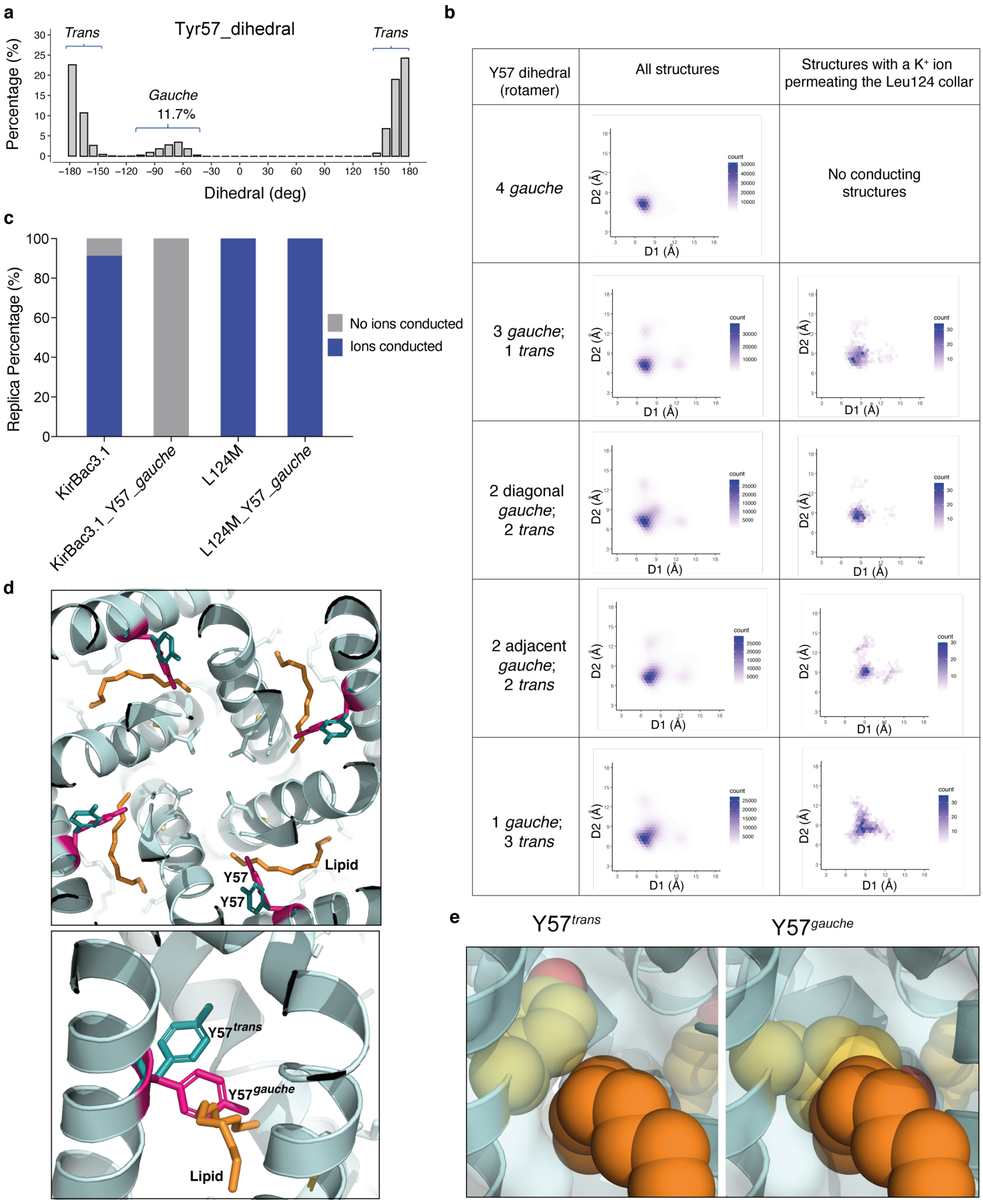

Supplementary Figure 6. The *gauche* rotamer of Tyr57 blocks lipid entering the fenestrations. (**a**) The proportion of the *gauche* conformer in the natural population, estimated by extracting data from all simulation structures, is close to 12 %. (**b**) Hexbin plots (as in Fig. 2d) showing the effect of excluding lipid from fenestrations on pore aperture. Notably, when all four Tyr57 adopt a *gauche* rotamer the collar remains small and impassable. (**c**) Quantification of ion conduction of simulation replicates of wild type KirBac3.1 and the L124M mutant when fenestrations are either untouched or blocked (Y57 *gauche*). (**d**) The *trans* rotamer of Tyr57 (deep cyan) allows lipid (orange) to enter the fenestrations, whereas the *gauche* rotamer (magenta) blocks the fenestration and lipid. (Top) Viewed along the molecular 4-fold axis (*z*), perpendicular to the membrane. (Bottom) Viewed from within the plane of the membrane. (**e**) A similar view of the fenestration entrance, this time with a transparent molecular surface, illustrates how Y57 *gauche* rotamer blocks the fenestration. In crystal structures (left) Tyr57 adopts *trans* rotamers, whereas in the Y57-gauche MD simulation model (right) it adopts *gauche* rotamers. Tyr57 is represented by yellow and red spheres. The alkyl chain from the crystal structure is represented by orange spheres (left), and is transposed onto the superimposed MD simulation model (right).

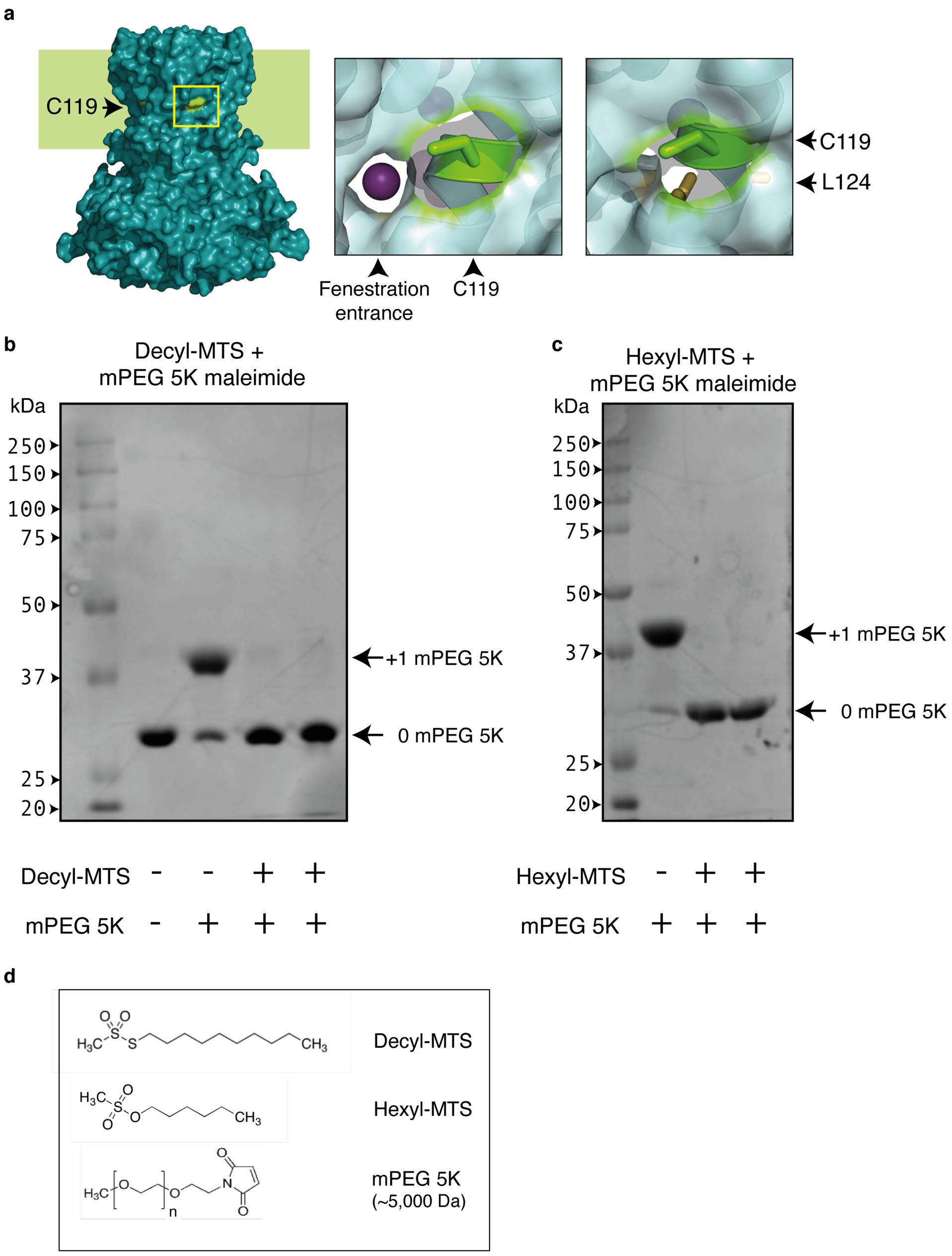

Supplementary Figure 7. MTS-alkyl derivatization. (**a**) The position of Cys119 (C119; chartreuse; in a yellow box) relative to fenestrations near the midpoint of the membrane is depicted by a molecular surface representation of KirBac3.1-SCS; the membrane region is shaded. Middle panel shows the spatial relationship of C119 to the fenestration entrance, aligned to show an internal K^+ ion through the fenestration; Right-hand panel shows the proximity to Leu124 The molecular surface immediately covering Cys119 is omitted for clarity. (**b**) Gel-shift assay used to indicate completeness of formation of C119-decyl by MTS-derivatisation of KirBac3.1-SCS, comparing the relative migration of unreacted (denatured; monomeric) KirBac3.1 with that of KirBac3.1 reacted with 5 mM methoxy PEG maleimide 5K (mPEG 5K) and KirBac3.1 derivatized with decyl-MTS prior to exposure to 5 mM methoxy PEG maleimide 5K. The shift in the band migration in lane 3 corresponds to the addition of a single 5K PEG per subunit of KirBac3.1. The absence of a band shift in lanes 4 and 5 indicates decyl-MTS has reacted with Cys119. The gel is representative of 6 independent experiments. (**c**) Gel-shift assay for hexyl-MTS derivatization of KirBac3.1-SCS, comparing the relative migration of KirBac reacted with 5 mM methoxy PEG maleimide 5K and KirBac derivatized with decyl-MTS before exposure to 5 mM methoxy PEG maleimide 5K. The gel is representative of two independent experiments. (**d**) Chemical formulae as indicated.

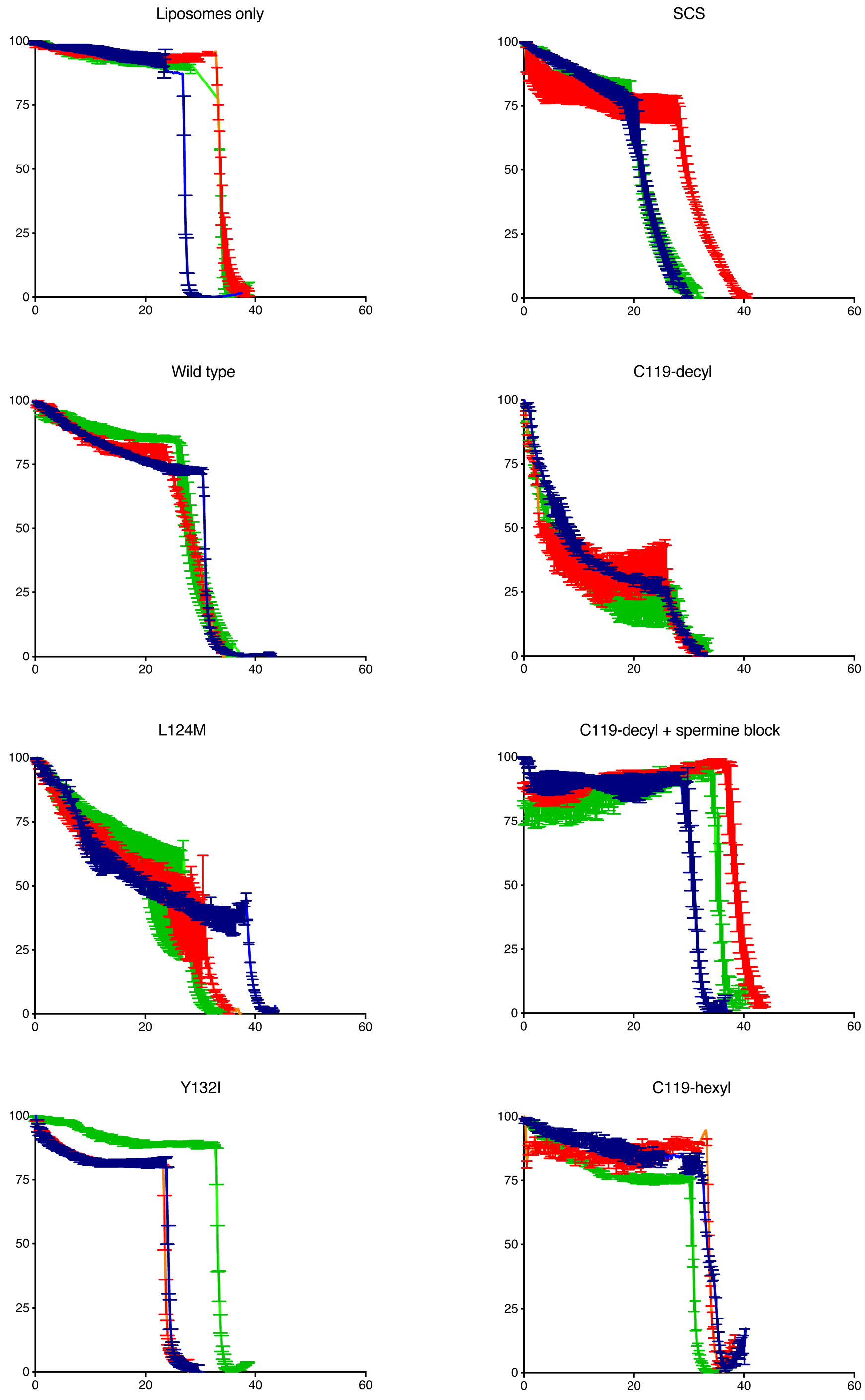

Supplementary Figure 8. Normalised data from liposomal fluorescence flux (ACMA) assays.

Three independent replicates are shown for each experimental condition, indicated by the title above each plot. For each experiment, each colour (red, blue, green) represents an independent reconstitution. The standard deviations from the mean (of three technical replicates) are indicated by error bars.

a

	Po	Ps	Po/Ps	Pburst	Rate O->S (ms^{-1})	Rate S->O (ms^{-1})	Io (pA)	Is (pA)	Vm (mV)	Is/Io
C119-Decyl mean, n=4	0.55	0.24	2.92	0.69	0.41	0.76	-3.18	-1.25	-80	0.393
sem	0.08	0.05	1.07	0.06	0.15	0.15	0.35	0.23		0.029
C119-Octyl n=1	0.36	0.41	0.89	0.46	1.25	1.07	-3.56	-0.76	-86	0.213
C119-Hexyl n=1	0.25	0.36	0.71	0.41	0.84	0.64	-3.20	-1.3	-80	0.510

b **c**

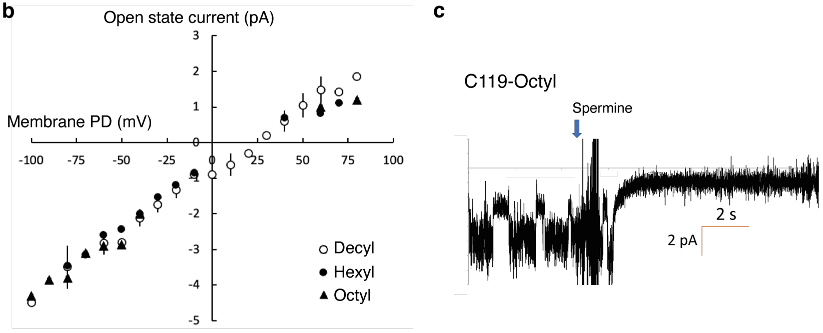

Supplementary Figure 9. HMM analysis of single channel recordings of C119-alkylated KirBac3.1. (**a**) Kinetic analysis, where I = current (pA), V = voltage, Po = probability of being fully open (maximal conductance), Ps = probability of being in a substate (lower conductance), Pburst is Po/(Po+Ps), the probability of being in the fully open state within a burst of activity, k = rate constant between fully open and substates. (**b**) Current-voltage relationship shows that aside from the differences in kinetics, the three derivatised species are fundamentally similar. Data are presented as mean ± SEM where applicable. The n values for decyl, hexyl and octyl are 4, 1 and 1 respectively. (**c**) Spermine sensitivity of C119-octyl at -80 mV.

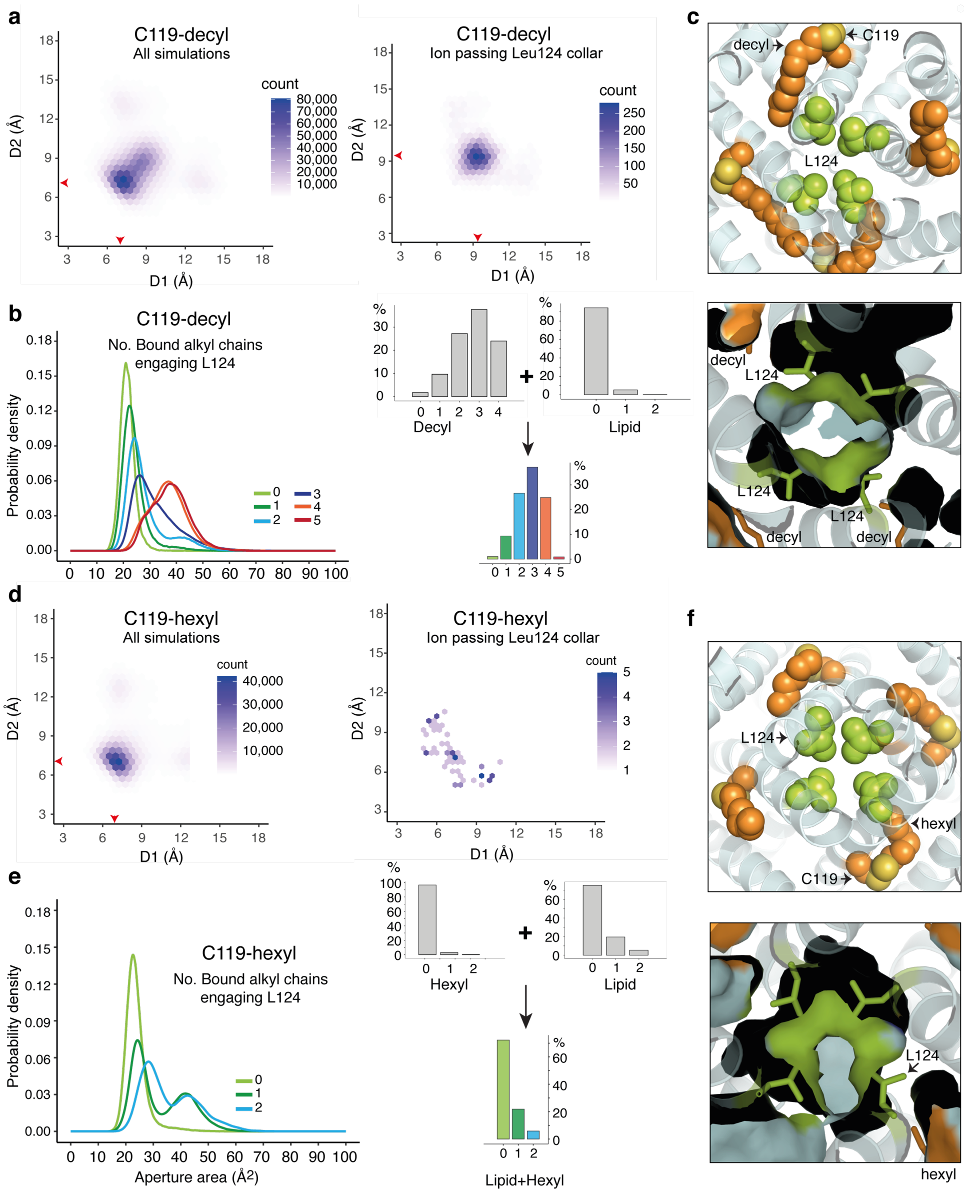

Supplementary Figure 10. Aliphatic chain occupancy affects pore aperture in the C119-derivatised channels similarly to the effect of lipids on wild type. C119-decyl and C119-hexyl substituents engaging Leu124, and the effect on pore aperture. (**a**) Hexagonal bin plots analysing the aperture diagonals in structures extracted from 70 simulations of unrestrained MD of C119-decyl. Distances are between centres of the red dots (midway between Cδ1 and Cδ2) in the schematic in Fig 2d. Red arrows on the plots indicate the population of highest density. (Left) Analysis of all structures. (Right) Analysis of structures in which a K^+ is passing the collar (**b**) The number of alkyl chains engaging with Leu124 side chains is correlated to the cross-sectional area of the 'open collar' between leucine side chains. (Right) In the C119-decyl channel, total alkyl occupancy can be contributed to by membrane lipids pushing in. (**c**) Transverse slice through the C119-decyl mutant at the Leu124 collar (yellow-green) of a random simulation structure with three fenestrations in which the occupying decyl substituents (orange) are engaging Leu124 residues. The accessible molecular surface below demonstrates the effect on the steric leucine cluster. (**d**) As for panel A*,* but hexbin plots are for C119-hexyl. Twenty simulations were carried out. (**e**) As for panel *b* but showing C119-hexyl distribution (**f**) Transverse slice through the C119-hexyl mutant as for panel C, shows one of four occupying hexyl substituents is reaching in far enough to contact a Leu124 residue (lower right corner)- the probability of this is only ~3 %. The accessible molecular surface below shows the sole leucine-hexyl contact allows limited opening of the steric cluster.

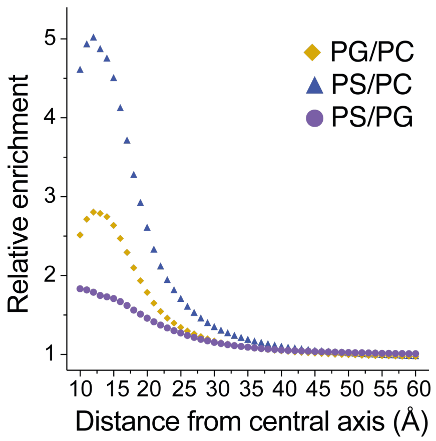

Supplementary Figure 11. Anionic lipids preferentially interact with the KirBac3.1 channel. Pairwise cross comparison of the probability density distribution of PC, PS and PG lipid head groups surrounding the pore derived from the coarse grained simulation data, showing that (on average) both anionic lipids are more closely associated with the channel than PC.

Supplementary Table 1. Crystallographic Structure Determination and Refinement.

Data collection	SCS<br>KirBac3.1_C71S-C119-C262S	L124M<br>KirBac3.1_C71S-C119-C262S_L124M
Processing system	XDS	XDS
Space group	$P42_12$	$P42_12$
Monomers per asymmetric unit	1	1
Cell dimensions (Å)	*a* 106.884, *c* 89.760	*a* 106.577, *c* 89.597
Wavelength (Å)	0.9537	0.9537
Resolution range (Å)	47.80/2.40	47.663/2.708
Observed/Unique reflections	276,325/20,993	190,560/26,392<br>(Friedel pairs)
R_{merge} (overall/outer shell) [a]	0.235/2.425	0.158/1.305
I/σI (overall/outer shell)	8.60/0.52	10.52/1.35
Completeness (overall/outer shell)	99.8 /99.3	99.8/99.2
Redundancy (overall/outer shell)	13.16/11.84	7.22/6.86
$CC_{1/2}$ (overall/outer shell)[b]	99.8/40.4	99.8/57.9

Data refinement		
Program	PHENIX	PHENIX
Resolution range (Å)	47.80 – 2.40	42.08-2.723
Reflections (refine/test)	20,950/1,050	14,342/700
R factor (R_{cryst}/R_{free})	0.2281/0.2583	0.2197/0.2507
Wilson B-factor	50.2	61.2
Mean isotropic *B*-factor B_{iso} ($Å^2$)	55.7	60.1
RMSD Bond lengths (Å)	0.002	0.002
RMSD Bond angles (°)	0.51	0.48
Ramachandran outliers	0	0
Rotamer outliers	3 (1%)	3 (1%)
PDB code	7N9L	7N9K

[a] $R_{merge} = \sum | I_{obs} - I_{calc} | / \sum I_{obs}$

[b] $CC_{1/2}$ = percentage of correlation between intensities from random half-datasets. Outer shell values are significant at the *p*=0.1% level.

Supplementary Table 2. Summary of Molecular Dynamics Simulations.

Equilibration		
Channel condition		Simulation length (ns)
Wild Type		101.5
Y132I		101.5
L124M		101.5
L124M: 4 *gauche*		101.5
Y57: 4 *gauche*		101.5
Y57: 3 *gauche*; 1 *trans*		101.5
Y57: 2 diagonal *gauche*; 2 *trans*		101.5
Y57: 2 adjacent *gauche*; 2 *trans*		101.5
Y57: 1 *gauche*; 3 *trans*		101.5
C119-decyl		101.5
C119-hexyl		101.5
Steered Molecular Dynamics Pulling		
Channel condition	Pulling Speed (Å/ns)	Simulation length (ns)
Wild Type	0.2	150
Y132I	0.2	120
L124M	0.2	110
Y57: 4 *gauche*	0.2	110
Umbrella Sampling		
Channel condition	Windows number * Simulation length per window (ns)	Simulation length (ns)
Wild Type	74*310	22,940
Y132I	97*200	19,400
L124M	53*310	16,430
Y57: 4 *gauche*	52*310	16,120
Unrestrained MD		
Channel condition	Simulation number * Simulation length (ns)	Simulation length (ns)

Wild Type	70*200	14,000
C119-decyl	70*200	14,000
C119-hexyl	20*200	4,000
Y57: 4 *gauche*	20*200	4,000
Y57: 3 *gauche*; 1 *trans*	20*200	4,000
Y57: 2 diagonal *gauche*; 2 *trans*	20*200	4,000
Y57: 2 adjacent *gauche*; 2 *trans*	20*200	4,000
Y57: 1 *gauche*; 3 *trans*	20*200	4,000
L124M: 4 *gauche*	20*200	4,000
Total simulation length (ns)		151,718
**Coarse Grained Simulations**		
Channel Condition	Simulation length (ns)	Simulation number * Simulation length (ns)
Wild Type	5*20000	100,000
Total coarse grained simulation length (ns)		100,000

Supplementary Table 3. Summary statistical data on fluorescence change for functional ACMA assays for control, wildtype, mutant, and C119-alkyl KirBac3.1. Data analysis was by two-sided t-test analysis of variance and refers to pairwise comparisons to KirBac3.1 or KirBac3.1-SCS as indicated (ns=non-significant; * p ≤ 0.05; ** p ≤ 0.01; *** p ≤ 0.001; **** p ≤ 0.0001). Dunnett's test was used to adjust for multiple comparisons to each control. 95% Confidence intervals (CI) are shown.

Condition	Mean fluorescence change prior to valinomycin addition ± SEM	Independent replicates (technical replicates for each experiment)	Significance (Sig) compared to KirBac3.1			Significance (Sig) compared to KirBac-SCS		
			Sig	P-value	95% CI	Sig	P-value	95% CI
Liposomes only (channel free)	7.74 ± 0.81	10 (5, 3, 3, 5, 4, 4, 4, 4, 3, 3)	****	<0.0001	15.28 to 25.00	****	<0.0001	17.07 to 26.86
Wildtype KirBac3.1	27.99 ± 2.65	6 (5, 3, 4, 3, 3, 3)				ns	0.6941	-8.707 to 12.36
KirBac3.1-SCS (*i.e.* C71S, C262S)	29.71 ± 3.34	3 (3, 3, 3)	ns	0.6941	-8.707 to 12.36			
L124M	55.71 ± 6.30	5 (3, 3, 3, 3, 3)	**	0.0019	13.36 to 42.30	*	0.0249	4.599 to 47.40
Y132I	16.81 ± 1.72	3 (3, 3, 3)	*	0.0289	-20.63 to -1.521	*	0.0265	-2.465 to -23.34
C119-Decyl	77.22 ± 2.36	3 (6, 3, 3)	****	<0.0001	39.46 to 59.21	****	<0.0001	-62.76 to -32.27
C119-Hexyl	16.86 ± 3.61	5 (3, 3, 3, 3, 3)	*	0.0329	-20.93 to -1.115	ns	0.0649	-0.7880 to 26.48
C119-Decyl + Spermine block (500 μM / 1000 μM)	5.56 ± 4.12	3 (3, 6, 6)	**	0.0021	-33.50 to -11.15	**	0.0037	8.909 to 39.40

Supplementary Table 4. Summary rate data for ACMA assays. Data analysis was by two-sided-t-test analysis of variance and refers to pairwise comparisons to KirBac3.1 or KirBac3.1-SCS as indicated (ns=non-significant; * p ≤ 0.05; ** p ≤ 0.01; *** p ≤ 0.001; **** p ≤ 0.0001). Dunnett's test was used to adjust for multiple comparisons to each control.

Condition	Mean rate (time constant, tau) ± SEM (min)	Independent replicates (technical replicates for each experiment)	Significance (Sig) compared to KirBac3.1			Significance (Sig) compared to KirBac-SCS		
			Sig	P-value	95% CI	Sig	P-value	95% CI
Wildtype KirBac3.1	20.4 ± 2.34	6 (5, 3, 4, 3, 3, 3)				ns	0.8041	-9.573 to 12.19
KirBac3.1-SCS	19.1 ± 3.23	3 (3, 3, 3)	ns	0.8042	-9.573 to 12.19			
L124M	19.6 ± 2.89	5 (3, 3, 3, 3, 3)	ns	0.8580	-8.510 to 10.13	ns	0.9269	-11.74 to 10.74
Y132I	11.9 ± 7.49	3 (3, 3, 3)	ns	0.1198	-2.404 to 19.36	ns	0.2480	-5.399 to 19.74
C119-Decyl	5.22 ± 1.2	3 (6, 3, 3)	**	0.0087	4.296 to 26.06	*	0.0322	1.302 to 26.44
C119-Hexyl	15.10 ± 3.23	5 (3, 3, 3, 3, 3)	ns	0.2786	-4.631 to 15.24	ns	0.4867	-7.762 to 15.75
