# Supplementary material for: Ion currents through Kir potassium channels are gated by anionic lipids
Source: Nat Commun. 2022 Jan 25;13:490. doi: 10.1038/s41467-022-28148-4 (PMC8789855; doi:10.1038/s41467-022-28148-4)
Supplement: Supplementary file 3 — Description of Additional Supplementary Files [file 41467_2022_28148_MOESM3_ESM.pdf]

Description of Additional Supplementary Files

File name: Supplementary Movie 1

Description: Potassium ions pass the Leu124 collar after it has been widened by interaction of Leu124 residues and lipid tails. A 150 nsec simulation of an ion being conducted through the pore of KirBac3.1. Concomitant orthogonal views show lipid tails (pink) moving in and out of the fenestrations to engage Leu124 (yellow). The Leu124 collar widens and narrows during the simulation, regardless of whether an ion is close by. When an ion is in the vicinity, it passes only when the collar is sufficiently wide.
