# Supplementary material for: Ion currents through Kir potassium channels are gated by anionic lipids
Source: Nat Commun. 2022 Jan 25;13:490. doi: 10.1038/s41467-022-28148-4 (PMC8789855; doi:10.1038/s41467-022-28148-4)
Supplement: Supplementary file 5 — Reporting Summary [file 41467_2022_28148_MOESM5_ESM.pdf]

nature portfolio

Corresponding author(s): Dr. Jacqueline Gulbis, Dr Brian Smith

Last updated by author(s): Dec 16, 2021

Reporting Summary

Nature Portfolio wishes to improve the reproducibility of the work that we publish. This form provides structure for consistency and transparency in reporting. For further information on Nature Portfolio policies, see our Editorial Policies and the Editorial Policy Checklist.

Statistics

For all statistical analyses, confirm that the following items are present in the figure legend, table legend, main text, or Methods section.

n/a	Confirmed	
☐	☒	The exact sample size (*n*) for each experimental group/condition, given as a discrete number and unit of measurement
☐	☒	A statement on whether measurements were taken from distinct samples or whether the same sample was measured repeatedly
☐	☒	The statistical test(s) used AND whether they are one- or two-sided<br>*Only common tests should be described solely by name; describe more complex techniques in the Methods section.*
☒	☐	A description of all covariates tested
☐	☒	A description of any assumptions or corrections, such as tests of normality and adjustment for multiple comparisons
☐	☒	A full description of the statistical parameters including central tendency (e.g. means) or other basic estimates (e.g. regression coefficient) AND variation (e.g. standard deviation) or associated estimates of uncertainty (e.g. confidence intervals)
☐	☒	For null hypothesis testing, the test statistic (e.g. F, t, r) with confidence intervals, effect sizes, degrees of freedom and P value noted<br>*Give P values as exact values whenever suitable.*
☒	☐	For Bayesian analysis, information on the choice of priors and Markov chain Monte Carlo settings
☒	☐	For hierarchical and complex designs, identification of the appropriate level for tests and full reporting of outcomes
☒	☐	Estimates of effect sizes (e.g. Cohen's *d*, Pearson's *r*), indicating how they were calculated

Our web collection on statistics for biologists contains articles on many of the points above.

Software and code

Policy information about availability of computer code

Data collection	XDS, Kabsch (version Jan 31, 2020); CHARMM-GUI, version 3.2; GROMACS, version 2020.1; CHARMM36m; CGenff, version 3.0.1; Martini, version 2.2; VMD, version 1.9.4a55; Xcalibur version 4.1
Data analysis	PHASER (2.8.3); CCP4i (version 7.1.016); COOT (version 0.9); PHENIX (version 1.19.2); HOLE (hole2; version 2.2.005); Hidden Markov Model custom software; Prism version 8 (GraphPad); R, version 4.0.5; Grace, version 5.

For manuscripts utilizing custom algorithms or software that are central to the research but not yet described in published literature, software must be made available to editors and reviewers. We strongly encourage code deposition in a community repository (e.g. GitHub). See the Nature Portfolio guidelines for submitting code & software for further information.

Data

Policy information about availability of data

All manuscripts must include a data availability statement. This statement should provide the following information, where applicable:

- Accession codes, unique identifiers, or web links for publicly available datasets
- A description of any restrictions on data availability
- For clinical datasets or third party data, please ensure that the statement adheres to our policy

Data availability
Source data are provided with this paper. Atomic coordinates and structure factors generated in this study have been deposited in the Protein Data Bank with accession codes 7N9L [http://doi.org/10.2210/pdb7N9L/pdb] and 7N9K [http://doi.org/10.2210/pdb7N9K/pdb]. Molecular simulation, fluorimetric assay and electrophysiological data generated in this study are provided as source data and have been deposited on the Figshare server at [https://doi.org/10.6084/

m9.figshare.c.5752604.v1]. Mass spectrometry lipidomics raw data generated in this study have been deposited on the Figshare server at [https://doi.org/10.6084/m9.figshare.17197811.v1].

Field-specific reporting

Please select the one below that is the best fit for your research. If you are not sure, read the appropriate sections before making your selection.

☒ Life sciences ☐ Behavioural & social sciences ☐ Ecological, evolutionary & environmental sciences

For a reference copy of the document with all sections, see nature.com/documents/nr-reporting-summary-flat.pdf

Life sciences study design

All studies must disclose on these points even when the disclosure is negative.

Sample size	We used two-way ANOVA to analyse differences in relative activity of Kir channels, with a null hypothesis that functional mutants exhibit no significant difference in activity from wild type (KirBac3.1 or KirBac3.1-SCS) channels. The two variables tested against this hypothesis are whether the ion conduction pathway is constricted (it is constricted in KirBac3.1 but not constricted in L124M), and the effect of C119-alkyl chain length. No sample size calculation was performed, but the P-values <0.0001 (****) for negative controls (liposomal, measured concomitantly with every experiment, and spermine-block) relative to wild type, and the consistency between the wild type conduction controls, KirBac3.1 and KirBac3.1-SCS, provided evidence for distinguishing changes in channel activity. Under these conditions, with relatively compact distributions, n=3 data points (each averaged from 3 – 6 technical replicates) are sufficient to discriminate between non-conducting, conducting and enhanced conduction in population assays. This is borne out at the single channel level in electrophysiology experiments using the same preparations of proteoliposomes.
Data exclusions	No data was excluded.
Replication	Each independent experimental replicates involves a new reconstitution of channels into proteoliposomes. Technical replicates are repeat measurements from the same liposome batch, and for each individual independent replicate, 3 - 6 technical replicates were carried out. A negative control (liposomes-only) was measures alongside each experiment. No measurements were excluded. Specific information regarding the number of replicates in each condition can be found in Supplementary Table 3.
Randomization	No experimental and control group allocation was carried out and so no randomization was performed.
Blinding	No experimental and control group allocation was carried out and so no blinding was performed.

Reporting for specific materials, systems and methods

We require information from authors about some types of materials, experimental systems and methods used in many studies. Here, indicate whether each material, system or method listed is relevant to your study. If you are not sure if a list item applies to your research, read the appropriate section before selecting a response.

Materials & experimental systems

n/a	Involved in the study	
☒	☐	Antibodies
☒	☐	Eukaryotic cell lines
☒	☐	Palaeontology and archaeology
☒	☐	Animals and other organisms
☒	☐	Human research participants
☒	☐	Clinical data
☒	☐	Dual use research of concern

Methods

n/a	Involved in the study	
☒	☐	ChIP-seq
☒	☐	Flow cytometry
☒	☐	MRI-based neuroimaging
